# Computer-Enhanced Visual Learning Method to Teach Endoscopic Correction of Vesicoureteral Reflux: An Invitation to Residency Training Programs to Utilize the CEVL Method

**DOI:** 10.1155/2012/831384

**Published:** 2012-01-09

**Authors:** Michael Bauschard, Max Maizels, Andrew Kirsch, Martin Koyle, Tony Chaviano, Dennis Liu, Rachel Stork Stoltz, Evelyn Maizels, Michaella Prasad, Andrew Marks, David Bolnick

**Affiliations:** ^1^Children's Memorial Hospital, Chicago, IL 60614, USA; ^2^Emory University School of Medicine, Atlanta, GA 30322, USA; ^3^The Hospital for Sick Children, Toronto, ON, Canada M5G 1X8; ^4^The University of Chicago, Chicago, IL 60037, USA; ^5^Seattle Children's Hospital, Seattle, WA 98105, USA

## Abstract

Herein we describe a standardized approach to teach endoscopic injection therapy to repair vesicoureteral reflux utilizing the CEVL method, an internet-accessed platform. The content was developed through collaboration of the authors' clinical and computer expertises. This application provides personnel training, examination, and procedure skill documentation through the use of online text with narration, pictures, and video. There is also included feedback and remediation of skill performance and teaching “games.” We propose that such standardized teaching and procedure performance will ultimate in improved surgical results. The electronic nature of communication in this journal is ideal to rapidly disseminate this information and to develop a structure for collaborative research.

## 1. Introduction

The series of paper on urinary tract infection management in this electronic journal deal with controversies on the surgical management of children with vesicoureteral reflux. Herein, we address controversies in surgical management as could stem from a lack of standardized teaching of the procedure. We describe a new method to standardize teaching of correction of vesicoureteral reflux (VUR) by endoscopic injection.

It is accepted that the surgical managements of VUR include open versus endoscopic repair and that while the procedures for open surgery have been largely agreed and are successful, those for endoscopic injection show a range of success rates and so have become controversial [1]. We believe this range in reported success likely depends on the methods used. Furthermore, in order to improve the surgical success experienced by practitioners with lower success rates, we believe a structured approach to the performance of the procedure is needed. We believe this is the case because there has yet to be a paradigm accepted as a standard method to perform endoscopic correction. To this end, we have developed the method of computer enhanced visual learning (CEVL) to present such a standard method [2]. The CEVL method includes personnel training, examination, and documentation using static and interactive computer visuals and games which are accessed via the internet.

We believe this method of standardized procedure performance will ultimate in improved surgical outcomes. The electronic nature of this journal for communication is ideal to rapidly disseminate this information and additionally provide a structure for collaborative research. We use this forum to invite residency training programs to utilize the CEVL platform for teaching and collaborative educational and clinical research.

We have shown previously that the CEVL platform to teach orchiopexy is effective in residency training [3, 4]. The platform is described in more detail below, but in brief it provides web-accessed training that shows procedure performance as components and steps, a utility for Likert's scoring of skills and for structure of feedback and remediation. This application is intended to be studied by the resident trainee in order to improve his/her next case skill performance. Additionally, the method has recently shown effective to train mental concepts such as grading of pediatric hydronephrosis [5] and open surgical skills in obstetrics [6] and orthopedics [7].

Herein, we apply the CEVL method to show its application to supplement resident teaching of endoscopic injection therapy for VUR. Furthermore, we propose use of this method to develop collaborative educational and clinical research with residency training programs which is aimed to improve standardization in teaching this procedure.

### 1.1. CEVL Method Defined

Traditionally, endoscopic surgery for the correction of VUR has been named according to the technique of injection. For example, Dr. O'Donnell's STING emphasizes the use of Teflon to bulk the base of the ureteral orifice [8], Lackgren's pamphlet codifies the Q-Med injection method using DEFLUX [9], Kirsch's *HIT *emphasizes the strategy of DEFLUX injection in the midtunnel as revealed by hydrodistention (HD) [10]; and finally Kirsch's *double-HIT *emphasizes the value of a 2nd injection to bulk the ureteral orifice [11]. Other variations have since been suggested [12]. Most recently has been the introduction of the *Injekt *needle to standardize needle placement in the ureteral orifice [1].

Using the CEVL method, we organize the procedure as a checklist to assess satisfying the component tasks needed to be performed in order to achieve a functional ureteral valve whether this requires only a single subtrigonal injection (STING), a single submucosal injection into the ureteral tunnel (HIT), two submucosal injections into the ureteral tunnel (Double Hit), or additional injections. The CEVL method defines criteria to determine when completion of each component is satisfactory.

## 2. Materials and Methods

### 2.1. Creation of the CEVL Module

The authors have conferred, compiled their approaches to endoscopic injection to correct reflux, and assimilated their concepts as a single method to perform endoscopic injection therapy using DEFLUX.

### 2.2. CEVL Method and Strategy of Surgery

The CEVL method to reconstruct the ureteral valve function is done by a series of injections wherein each injection “braces” the next injection. The procedure is done as a checklist to perform a series of hierarchical components each of which involves injection of implant while assessing the morphology of the ureter orifice and/or submucosal ureteral tunnel. The injections are accomplished by having the surgeon draw specific skills from a skills bank including injecting minimally or slowly, hard or soft flush, and tenting the mucosa during injection.

The strategy for the procedure is to reconstruct the malformed ureter orifice as follows.

1. Elevate the ureter orifice. *Pathology*. A Lax trigonal muscle complex permits the normally elevated ureteral orifice (“A” configuration) to recede to the level of the bladder mucosa and foreshorten the submucosal tunnel. *Treatment*. Injection therapy creates a “nipple” valve effect by elevating the orifice on a “mesa” so that the tunnel is effectively lengthened. In other words, the higher the mesa, the longer and more effective are the nipple and antireflux valve.
2. Reconfiguration of the ureteral meatus as a slit. *Pathology*. Laxity of the longitudinal muscle coat of the ureter causes the ureteral orifice to become patulous and thereby permit reflux. *Treatment*. Implant injected into the submucosa causes apposition of the mucosal surfaces thus creating a slit-like morphology which prevents reflux as it now does not show hydrodistention.

In summary, the longer the submucosal tunnel length which is compressed by bladder hydrostatic pressure, the more effective the flap valve becomes against reflux; similarly the higher the ureteral orifice, the more effective the nipple valve against reflux. In this manner as bladder pressure increases with filling or voiding, the pressure burden is applied along the length of the nipple (height of the mesa) which constricts the channel and prevents reflux, and the slit meatus morphology restricts reflux. Thereby correction of these two features is the basis for the creation of a new anti-reflux mechanism.

While the CEVL method is regarded as a template for procedure performance, it is amenable to deviations in two ways as follows. First, individual ingenuities may need to be applied intraoperatively, such as cases in which a ureteral orifice is not aligned with the cystoscope and injection becomes difficult (e.g., an assistant may need to push the ipsilateral side of the partially filled bladder to “move” the ureteral orifice closer to the axis of the injection needle). Second, CEVL can be customized to show an individual surgeon's preferences which may differ from the template procedure.

### 2.3. CEVL Module

The module is organized to show preparations which need to be done (i.e., by operating room staff, surgeon, and for the patient), performance of the procedure by hydrodistention grading system (HD grade 0–3), learning aids, and feedback and remediation of skills.

## 3. Preparations

Goals for each type of personnel in the operating room are presented as a checklist.

### 3.1. Patient Preparations' Checklist

In the outpatient area, consent should be obtained from the patient after all relevant concerns are discussed for their particular case, and the site(s) targeted for repair is marked on the patient. Prior to induction of anesthesia, the patient's urine should also be checked for sterility. While it may seem mundane to confirm the urine is sterile, many of the patients who are considered for antireflux surgery have had multiple infections. Since injections may seem normal to the child, the family may be unaware of this problem. This test is crucial to avoid injection of a foreign substance in the face of an active infection. Although this remains a concern only theoretically, it is best to have the knowledge of infection prior to starting the surgical procedure.

After anesthesia is induced, the patient is positioned in the modified lithotomy position to assure endoscopic access to ureters which may be extremely laterally ectopic. Bundling any cystoscopy, video camera, light cords, and position of the video screen were detailed. Agreement on how placing the light cords on “standby” when not in use is achieved with the OR staff.

### 3.2. Surgeon Preparations' Checklist

The surgeon first prepares for surgery by reviewing CEVL. This may be as an introduction to the procedure for residents who are in training or as an attending, as a memory refresher. The surgeon's checklist begins with a review of the CEVL injection therapy module. This includes reviewing the module, showing their “readiness” through the interactive quiz, and reviewing the procedure components and steps. Next, the surgeon will devise and understand the procedural strategy for the case. This is done by first viewing the normal trigone, then comparing this appearance of a normal ureteral meatus to each HD grade. The surgeon will then “huddle” with the attending to agree on grade of VUR, address expected deviations from the CEVL module, review typical rescue strategies, and check the integrity of the endoscopic instruments/injection needles and availability of suitable amounts of DEFLUX. CEVL visuals show 3D presentations of the injection needle and its protrusion from the open end of the cystoscope.

### 3.3. Room Preparations' Checklist

CEVL stresses teamwork by defining team techniques. Teamwork is introduced by recognizing the operating room personnel names and agreeing on duties of these members. This establishes the responsibilities of each staff member. A room “chalk outline” is provided to show the expected position of equipment (e.g., video tower/screen), the personnel (e.g., scrub tech assistance with DEFLUX), and the patient. Light cord function, table controls, irrigation, and nuances such as OR noise level are all considered. Although these may seem trivial, these instructions are necessary to increase efficiency and prevent errors. Personal needs of the surgeon including pager, glove size, and hand washing are addressed, as few details are overlooked. The team technique of “time out” is utilized to confirm availability of enough DEFLUX to inject (at least two syringes/ureter) or fluoroscopy if intraoperative cystography is anticipated.

The *operating room nursing staff *may access the CEVL platform to educate themselves and understand the expected routine for surgery performance. Here they learn the instruments to be used, the instrument names colloquial to the operating room, and where they are placed on the cystoscopy back table.

Similarly, *anesthesiologists *may access the module in order to prepare for routines they will be expected to perform which are specific to the procedure. For example, after general anesthesia is induced, it is a surgical routine to request raising the table to accommodate cystoscopy viewing using the cystoscope camera. Another example includes the need for the anesthesiologist to move the child towards the foot of the bed in order to allow full range of movement for cystoscopy. In this simple manner, the anesthesiologist can anticipate performing these procedures and thereby integrate such needs into his/her routine work flow.

*Radiology technicians *can learn the surgical procedure and the reason for the need for intraoperative X rays. Furthermore, they may access the room chalk outline to better anticipate where the equipment needed is to be placed in the room.

This section concludes with the completion of a readiness quiz as a method to verify the staff's awareness of the needs they will be required to perform during the case.

## 4. Procedure

The checklist items for the procedure are shown (Table 1).

### 4.1. Inspection of Bladder and Ureter Orifice

**Figure 1:**
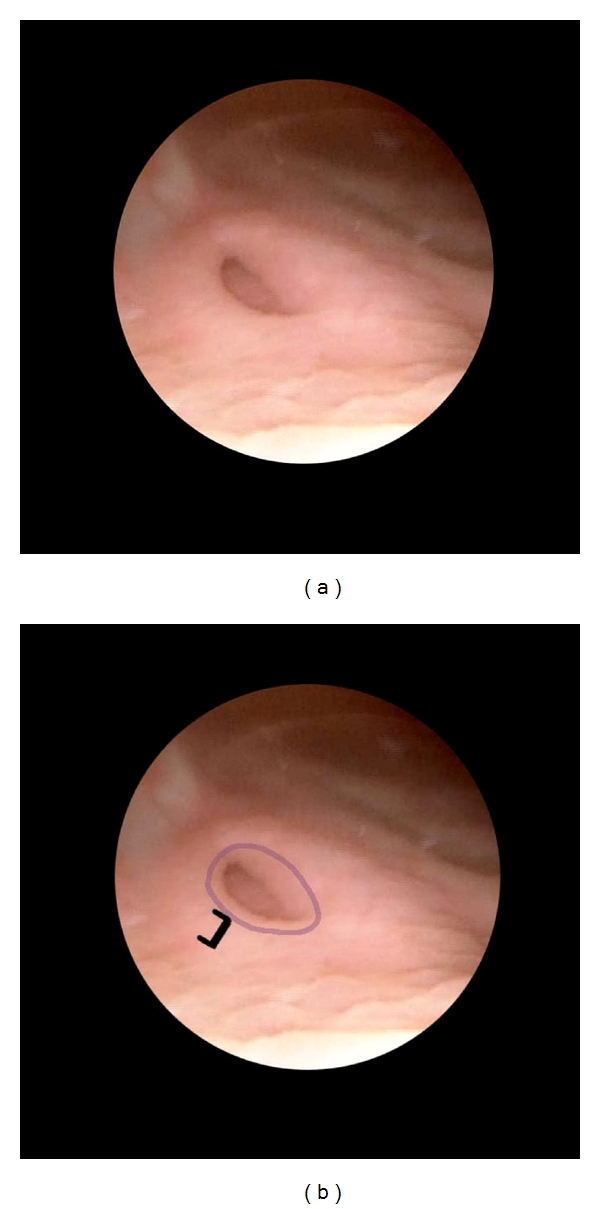
Procedure as CEVL components in a case of grade IV reflux in an infant girl. Inspect the ureteral orifice. The ureteral orifice is identified. It is patulous (blue ring) laterally positioned (not evident in this close-up view) and is almost flush with the bladder surface (as indicated by the graduated rule). It shows HD3 (not shown). In selected images which follow, this image is used as a reference for the *original *appearance of the orifice before reconstruction.

- It is implicit that a thorough examination of the genitalia is performed prior to endoscopic surgery. Such examination may reveal associated abnormalities that could interfere with the endoscopic procedure such as meatal anomalies in boys (e.g., stenosis/hypospadias) or ureteral anomalies in girls (e.g., ectopic ureter).
- Passage of the pediatric cystoscope along urethra and into bladder. Special techniques for passing into infant male and female urethras are addressed. For example, the technique of lifting and laterally spreading the labia majora to identify and enter the urethral meatus in a young female is shown. After entering the bladder, the trigone can be visualized such that following the trigonal ridge aids identifying the ureteral orifices.
- The HD grades of the orifices are assessed. Assessing ureter hydrodistention grade is started with the bladder underfilled so that a hard flush will hydrodistend the orifice and thereby provide an open view of the tunnel. After a brief period, the tunnel will fully open and grading HD is done. This assessment of HD grade is used to predict the volume of implant to inject (Figure 1). The VCUG of the case is shown in Figure 6.

### 4.2. Needle Enters Ureter

**Figure 2:**
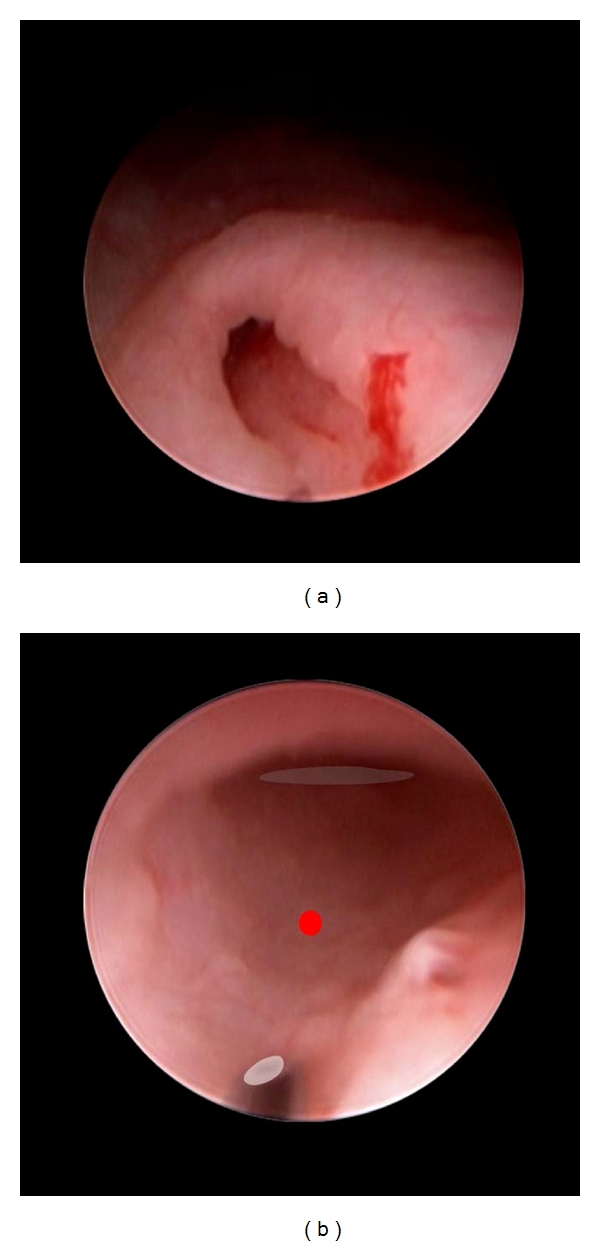
Procedure as CEVL components in a case of grade IV reflux in an infant girl. Enter the ureter orifice. (a) After hard/soft flushing of irrigation hydrodistends the ureter meatus such that the injection needle may enter tunnel. Alternative methods of entry are described in the electronically accessed module. (b) Needle tip (colored overlay) has entered tunnel and is positioned to inject at the halfway mark (red oval) between orifice (not shown) and detrusor hiatus (blue oval).

Injection needle is inserted into submucosal ureter after establishing the landmarks for insertion of the needle tip, the detrusor hiatus, and ureteral orifice (Figure 2). *Needle tip placement is at the midpoint between these two landmarks, on the tunnel floor *(i.e., the 6 o'clock position)*, and the bevel is “up,” *facing the lumen. Passage of the needle tip beyond the hiatus will result in the pelvic ureter rather than submucosal ureter injection. This component is finished when the needle tip is placed correctly on the floor of the tunnel.

### 4.3. Midtunnel Injection

**Figure 3:**
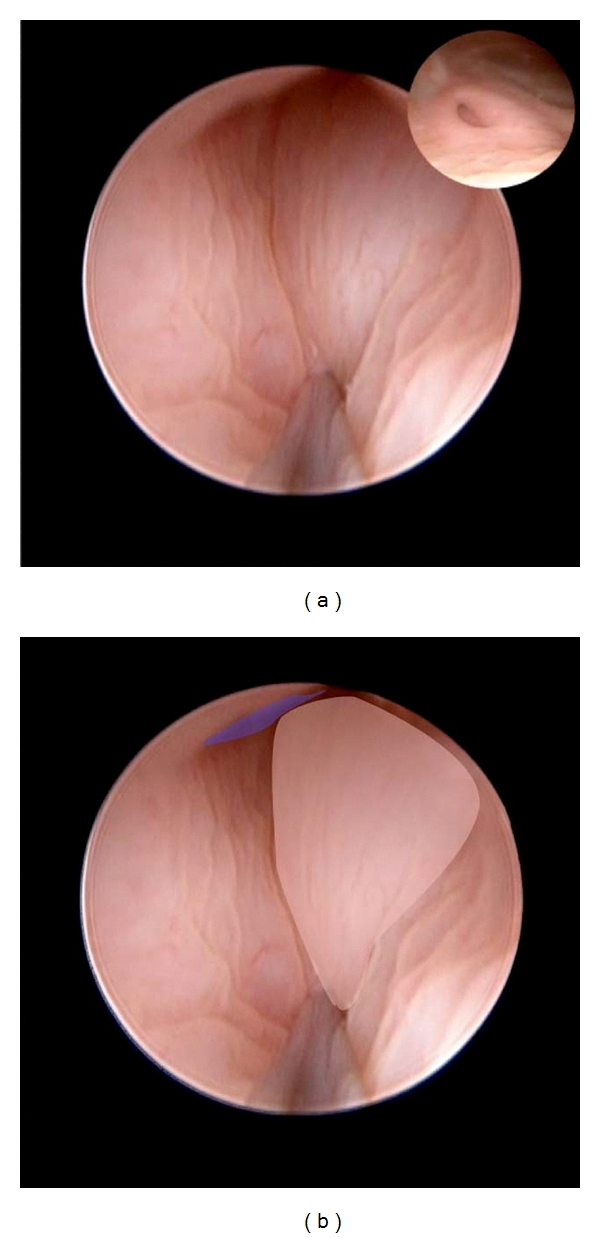
Procedure as CEVL components in a case of grade IV reflux in an infant girl. Mid-tunnel injection. Mid-tunnel injection is positioned as in Figure 2. The injection shown here has bulked the submucosa (overlay right). This component is complete as only a crescent of the ureteral lumen is still evident (blue overlay).

- With the needle positioned as above and the needle indicator bar confirming needle bevel faces the lumen, the *needle is advanced *below the mucosa so that the black indicator ring is just deep to the entry point. This assures the needle is inserted just submucosally and the submucosa will fill properly.
- The *bladder is drained *by opening of the stop-cock, then a *soft flush *is performed while DEFLUX is *injected minimally, *as in the hydrodistention injection therapy (HIT) procedure. This should confirm that there is no leakage of DEFLUX (Figure 3). Next, as *slow Injection *is done, the implant spreads out to encircle the submucosal tunnel. While injecting, the needle may be pulled back as to pull the mucosa forward by *tenting *it thereby encouraging filling the orifice distally. This needle pull should be done cautiously to avoid dislodging the needle out of the mucosa. An *elevation *of the submucosa should be confirmed as rising from the floor or the sides of the mucosa. Injection continues slowly. This implant braces the next injection, distal tunnel injection. This component is finished when only a crescent of lumen can be seen, and the HD grade decreases from that grade before this injection began.

### 4.4. Distal Tunnel Injection

**Figure 4:**
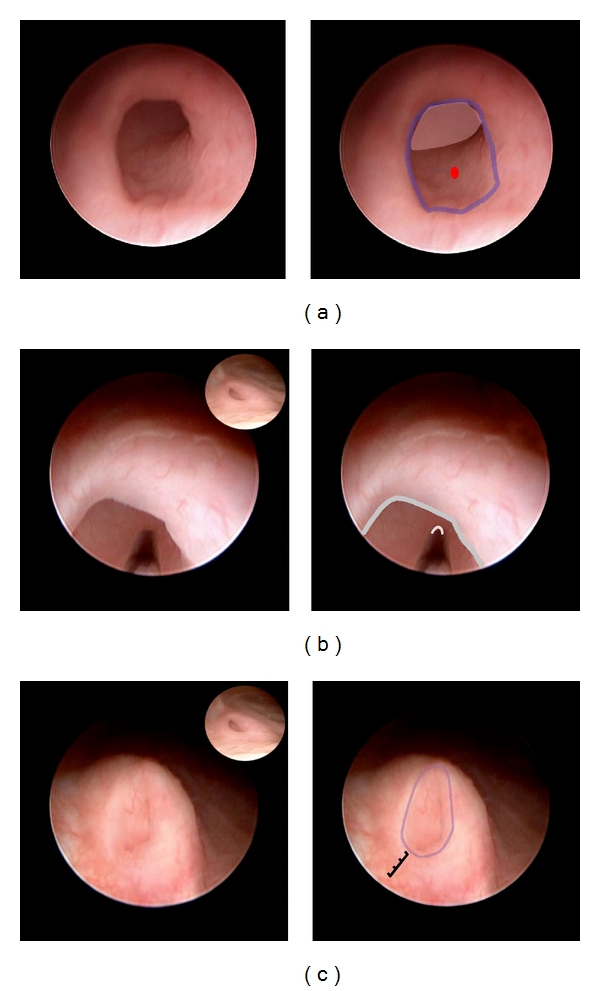
Procedure as CEVL components in a case of grade IV reflux in an infant girl. (a) Distal tunnel injection sequence. Injection site is judged as the halfway mark between ureter orifice (blue ring) and last injection in the background (translucent overlay). (b) Needle tip (left overlay) has penetrated mucosa and ready to inject implant. Orifice margin is noted (left overlay). (c) injection is completed as the mound of bulking implant (right overlay) obscures the lumen. The margin of the ureteral orifice is further elevated above the bladder surface (blue overlay) as shown by the graduated rule. This component is complete as the lumen beyond injection site is no longer evident and there is no evidence of HD.

- The distal tunnel injection site (as in the double HIT procedure) is at the halfway point between the newly formed midtunnel mound and the ureteral orifice. The injection is done as in the midtunnel injection. Distal tunnel injection causes the orifice to rise somewhat on the floor of the bladder (Figure 4). This component is completed when the lumen beyond injection site is no longer evident. And there is no evidence of HD (i.e., H0).

### 4.5. Orifice Injections

**Figure 5:**
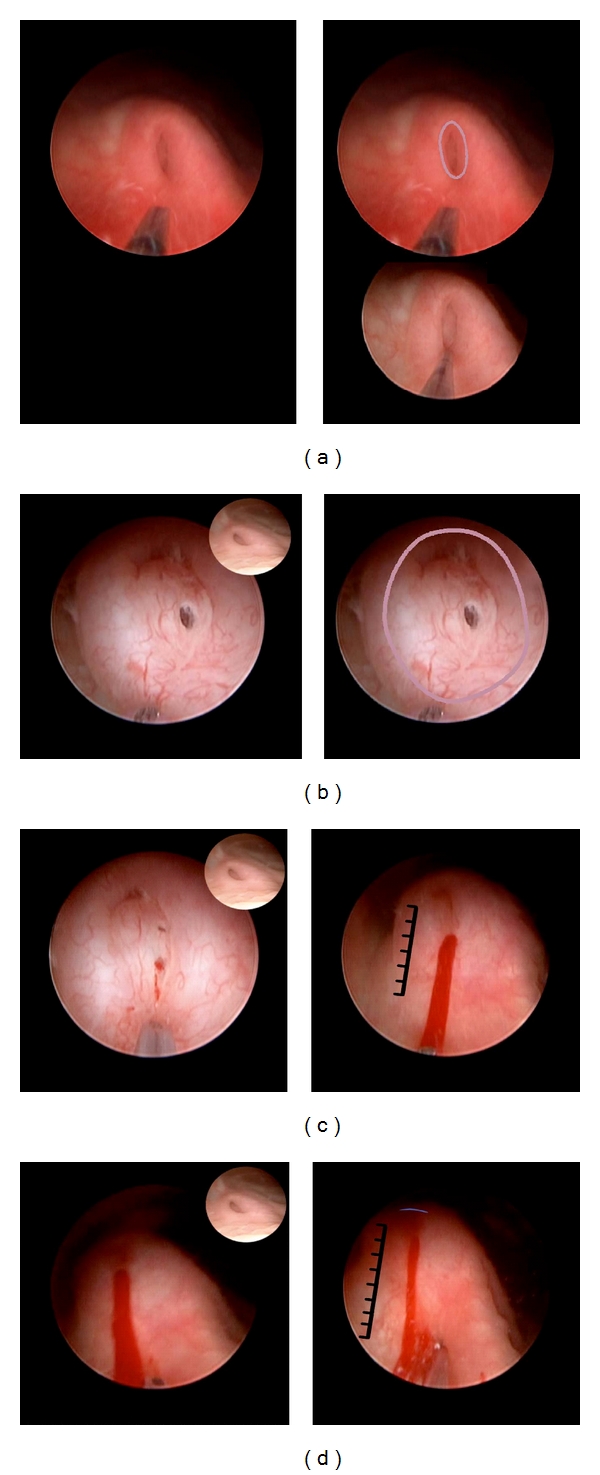
Procedure as CEVL components in a case of grade IV reflux in an infant girl. (a) Ureter orifice injection sequence. Inner margin of orifice (top left). Needle tip targets the injection site into the inner margin of ureter orifice (top right overlay). Bottom right: needle tip has penetrated inner margin of ureter orifice. (b) Injection at inner margin of orifice is now complete as the orifice is raised further above bladder surface. Hole in mucosa represents previous needle penetration. This component is complete as this appearance shows that the implant mound obscures view of the ureter lumen and has further elevated the ureter orifice. (c) Outer margin of ureter orifice sequence. Left: needle tip targets next injection at the outside margin of orifice. Right: after injection there is additional bulking of the orifice which is further elevated (graduated rule). There is an insignificant blood stream from the site of previous injection. This component is complete as the implant injection further elevates the ureter orifice. (d) Base of ureter orifice injection sequence. (a) Needle tip targets injection site at base of mesa. (b) *After *injection. This component is now complete as the mesa is further elevated (graduated rule), ureteral meatus is slit-like (blue crescent), and the meatus does not show hydrodistention (not shown). This appearance is compared with the icon representing the orifice before reconstruction.

**Figure 6:**
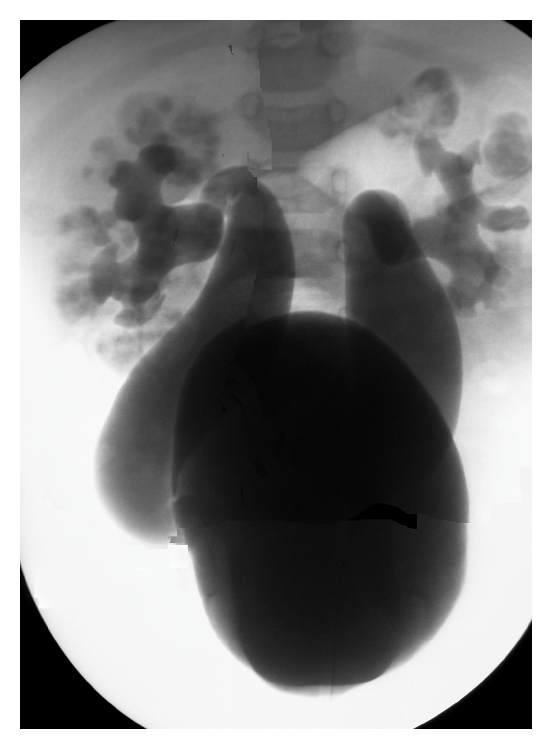
Procedure as CEVL components in a case of grade IV reflux in an infant girl. Voiding cystourethrogram (VCUG) showing Grade IV reflux in an 18 month old girl with recurrent febrile urine infection which breaks through prophylactic antiseptic administration. There is a family history or reflux. The family chose to repair reflux by endoscopic injection therapy. The CEVL method was used as is shown.

Injections into the orifice margins are done as needed (Figure 5). These are placed in the following locations.

- Inside margin. Injection at the inside margin of the ureteral orifice further elevates the orifice above the bladder surface. This component is complete when the implant mound obscures view of the ureter lumen and further elevates the ureter orifice.
- Outside margin is reserved for ureter orifices which show HD grade more extreme than “3” and require further elevation of the orifice by injection. This component is complete when the implant injection further elevates the ureter orifice.
- Base of orifice injection (as in the STING procedure) elevates the meatus higher above the bladder mucosa creating a nipple valve effect. The base of the “mesa” is where the formed mound meets the floor of the bladder. Further injection here will elevate the orifice substantively emulating the appearance of a mesa and creating a nipple. The ureter orifice is now typically elevated and the meatus appears as a slit.

This component is complete when the orifice is further elevated on the mesa, ureteral meatus is slit-like, and the meatus does not show HD.

The endoscopic injection procedure to correct reflux is completed having satisfactorily performed the components check listed. Orifices with lower grades of HD may require fewer injections.

### 4.6. Supplemental Injections

After completing the standard injections and reviewing progress made, additional, or supplemental, injections should be done if the standard set does not accomplish the treatment goals. They may be made into the medial/lateral pillars of the orifice or the submucosal roof.

### 4.7. Postoperative Appearances

CEVL shows the postoperative appearances of reconstructed orifices by ultrasound and endoscopy.

### 4.8. Special Cases

CEVL injection therapy for reflux involving additional anomalous ureteral orifice morphologies is shown: small orifice makes needle entry difficult, duplication, periureteral diverticulum, ureteral duplication and reflux, cystitis cystica, and reflux after open reimplantation. The most common special case encountered in clinical practice of endoscopic injection therapy is the orifice which, despite permitting reflux, is too narrow to easily admit the traditional injection needle. In this special case, utilization of specialized needles with filiform tips (INJEKT, Cook Urological) facilitate procedure performance.

### 4.9. Performing the Procedure in a Residency Training Hospital

Various educational tools are included in the module including computer interactives (i.e., pdf interactive) which allows users to manipulate a digital needle as a “game” for correct placement and rotation of the needle. A Readiness quiz is provided for trainees as a method to drive home the key points and to show to their attending and themselves that they are prepared to perform the surgery. OR staff may utilize the module to learn the procedure, instruments, and their role during surgery (e.g., act as a sentry checking for adequacy of irrigation fluid/video monitor needs). Feedback on procedure performance is inputted as Likert's scale ratings and free text. Structured remediation on skills is entered to guide improvements for “next case” performance.

## 5. Discussion

We have presented a new web application CEVL to promote standardization in teaching endoscopic injection therapy of pediatric VUR. Our anecdotal experiences in utilization of the module show it is well received by operating room staff, attendings, residents, and pediatric urology fellows (coauthors A. Marks and M. Prasad). We believe this is because the CEVL presentation of performance of the procedure explicitly using text with narration, pictures, and video as accessed by the operating room staff better integrates them into the procedure performance. The feedback and remediation application in CEVL permits residents to identify aspects of surgery most needy of remediation and thereby focus their practice in order to improve “next case” performance. CEVL provides administrative tools for residency program directors to monitor the progress of their trainees' skill acquisition towards mastery by review of their skill scores. Furthermore CEVL utilization would develop data sets to show a timeline for the expected rate of skill acquisition to perform the procedure in residency training and/or standards for proficiency in performance.

Additional benefits of CEVL utilization to residency training programs are described in the following sample scenarios. Attendings may structure content as web links which detail their differences in procedure performance so as to permit residents to anticipate how they expect to perform the procedure according to their individual attending supervisor. CEVL utilization facilitates resident-to-resident education in that CEVL enables upper-level residents to teach lower-level residents how to perform the surgery using their unique content. Clinicians after-residency may utilize CEVL to formulate a CEVL module or “CEV-ule” unique to their practice style, such that, when they complete their training, they may utilize the information at hospitals in which they are on staff to educate them on how to perform the procedure.

While many details have been included in this communication, accessing the complete module electronically will provide the intended learning experience. Therefore, we invite residency training programs to access this tool to promote teaching the procedure and to structure collaborative educational and clinical research. This may be done by contacting info@cevlforhealthcare.org. or visiting http://cevlforhealthcare.org/ (last accessed September 15, 2011).

We regard CEVL methods as a modern supplement to the late 1890's Halstedian method used to train performance of surgical procedures by immersion in the clinical arena, “as see one, do one, teach one.” While historically time honored and effective, it is generally now viewed that this method has become less efficient because of changes in surgical technology an administration of residency training. For example, new demands for increasing technical knowledge needed to perform recent innovative procedures (e.g., robotic surgery). Also the reduced time allocated for resident immersion in the operating room secondary to reduced resident duty hours restricts access to learning surgical skills [13]. From this background, we note that methods to train the performance of endoscopic injection therapy to repair pediatric VUR has changed from ad hoc training at local residency programs to personalized brief, one-day, training seminars [14]. To date there are no widely accessible organized methods to teach this procedure to large audiences.

We recognize that while there are published reports showing the effectiveness of endoscopic injection to correct reflux, they stem from selected centers which have chosen to focus utilization of this technique [15]. There is no documentation that the experience of this published literature reflects that of less experienced or even infrequent proceduralists. It is reasonable to ponder the number of procedures recommended as privilege to achieve a good experience on endoscopic treatment of VUR; however, as this paper deals primarily with teaching trainees the procedure, it remains for future research on objective assessment of skill proficiency needed to privilege procedure performance. From this perspective, we expect that the CEVL application is also suited for review by the proceduralist who is a novice or who performs this surgery only infrequently and so will likely desire improvement in his personal surgical success rate.

**CEVL as a Format to Teach Urological Surgery**

CEVL is an online tool used for instruction, training, and documentation of procedure performance. It has been used since 2003 and reported in 2008 [2]. The CEVL interface shows the user contextual information through the use of text, images, video, and audio narration. Interactive portions of the presentation require users to demonstrate their knowledge of procedure performance by satisfactorily responding to “ready for surgery” questions or by manipulating a digital needle injection for correct placement.

## 6. Conclusions

The CEVL method is a novel approach to teach and perform endoscopic injection therapy which may be utilized by the entire OR staff. We believe it helps residents and staff to prepare and perform surgery by providing a shared knowledge to all operating room staff which familiarizes them with the procedure and its intricacies. The method is also amenable to be utilized for collaborative educational and clinical research on endoscopic surgery for vesicoureteral reflux.

We invite urology residency training programs to participate in utilization of the module in order to gain further experience to teach and learn surgery and to perform collaborative educational and clinical research. Interested programs would contact info@cevlforhealthcare.org.

## Figures and Tables

**Table 1:** Checklist inventory of assessment of ureteral orifice reconstruction and desired end result for each component.

Check listed component	Satisfaction of end result
(1) Inspection	Assess orifice hydrodistention grade 0, 1, 2, or 3
(2) Enter tunnel	Hard/soft flush opens orifice
(3) Mid-tunnel injection	Only crescent of lumen remains
(4) Distal tunnel injection	Lumen is not evident
(5) Orifical injections	
(a) Inner orifice	Orifice is elevated above bladder surface
(b) Outer orifice	Orifice is further elevated above bladder surface
(c) Base of orifice	Orifice is further elevated above bladder surface
